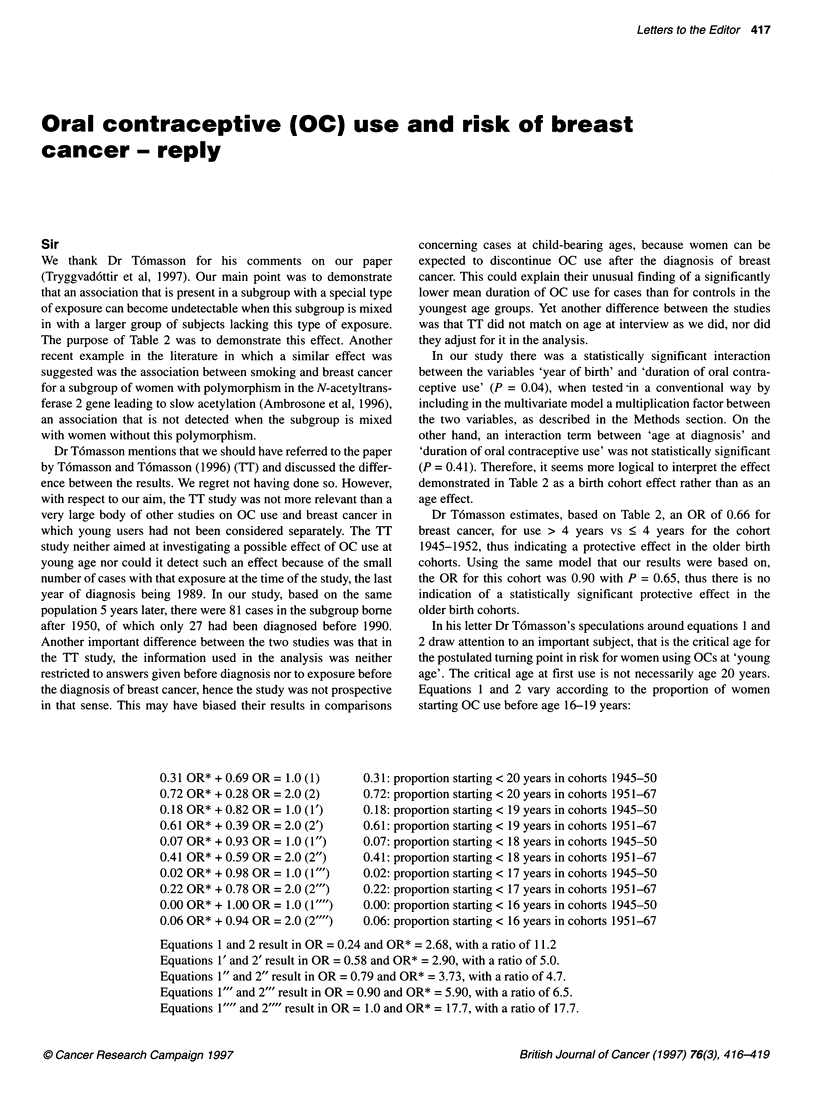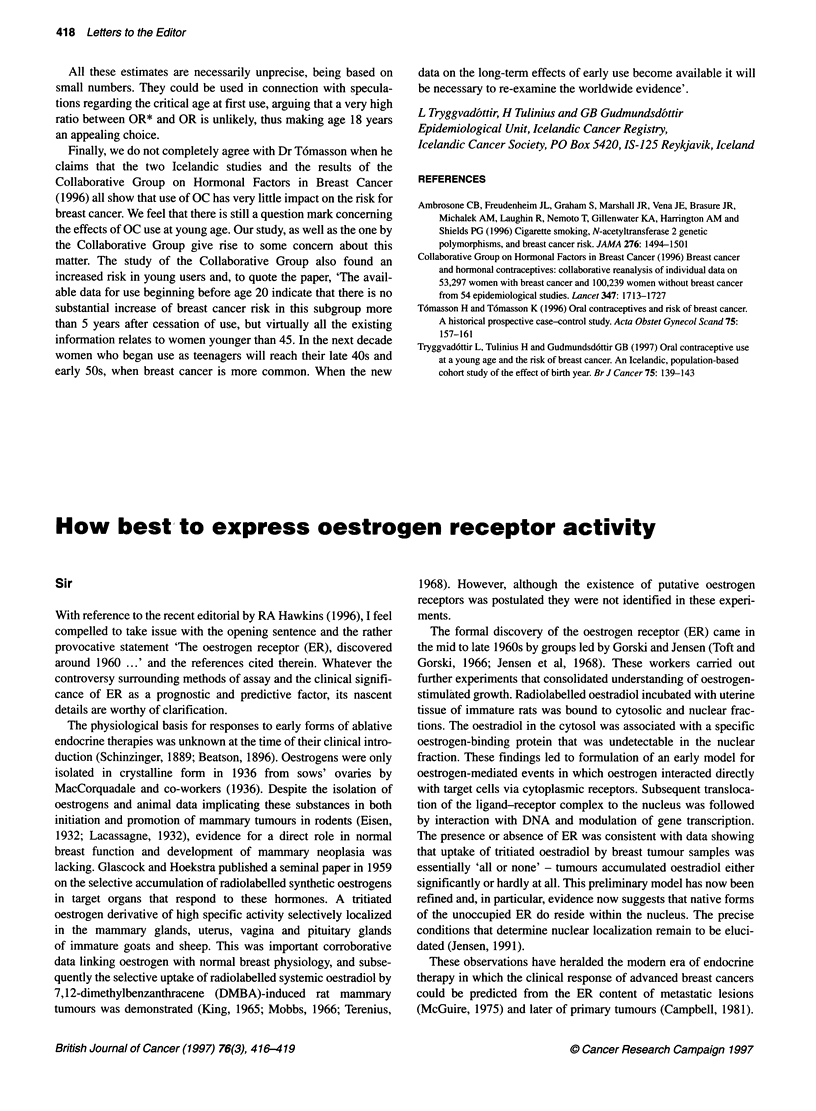# Oral contraceptive (OC) use and risk of breast cancer - reply

**Published:** 1997

**Authors:** L Tryggvadóttir, H Tulinius, GB Gudmundsdóttir


### Oral contraceptive (OC) use and risk of breast cancer – reply

**Sir**

We thank Dr Tómasson for his comments on our paper (Tryggvadóttir et al, 1997). Our main point was to demonstrate that an association that is present in a subgroup with a special type of exposure can become undetectable when this subgroup is mixed in with a larger group of subjects lacking this type of exposure. The purpose of Table 2 was to demonstrate this effect. Another recent example in the literature in which a similar effect was suggested was the association between smoking and breast cancer for a subgroup of women with polymorphism in the *N*-acetyltransferase 2 gene leading to slow acetylation (Ambrosone et al, 1996), an association that is not detected when the subgroup is mixed with women without this polymorphism.

Dr Tómasson mentions that we should have referred to the paper by Tómasson and Tómasson (1996) (TT) and discussed the difference between the results. We regret not having done so. However, with respect to our aim, the TT study was not more relevant than a very large body of other studies on OC use and breast cancer in which young users had not been considered separately. The TT study neither aimed at investigating a possible effect of OC use at young age nor could it detect such an effect because of the small number of cases with that exposure at the time of the study, the last year of diagnosis being 1989. In our study, based on the same population 5 years later, there were 81 cases in the subgroup borne after 1950, of which only 27 had been diagnosed before 1990. Another important difference between the two studies was that in the TT study, the information used in the analysis was neither restricted to answers given before diagnosis nor to exposure before the diagnosis of breast cancer, hence the study was not prospective in that sense. This may have biased their results in comparisons concerning cases at child-bearing ages, because women can be expected to discontinue OC use after the diagnosis of breast cancer. This could explain their unusual finding of a significantly lower mean duration of OC use for cases than for controls in the youngest age groups. Yet another difference between the studies was that TT did not match on age at interview as we did, nor did they adjust for it in the analysis.

In our study there was a statistically significant interaction between the variables 'year of birth' and 'duration of oral contraceptive use' ($P = 0.04$), when tested in a conventional way by including in the multivariate model a multiplication factor between the two variables, as described in the Methods section. On the other hand, an interaction term between 'age at diagnosis' and 'duration of oral contraceptive use' was not statistically significant ($P = 0.41$). Therefore, it seems more logical to interpret the effect demonstrated in Table 2 as a birth cohort effect rather than as an age effect.

Dr Tómasson estimates, based on Table 2, an OR of 0.66 for breast cancer, for use > 4 years vs ≤ 4 years for the cohort 1945–1952, thus indicating a protective effect in the older birth cohorts. Using the same model that our results were based on, the OR for this cohort was 0.90 with $P = 0.65$, thus there is no indication of a statistically significant protective effect in the older birth cohorts.

In his letter Dr Tómasson's speculations around equations 1 and 2 draw attention to an important subject, that is the critical age for the postulated turning point in risk for women using OCs at 'young age'. The critical age at first use is not necessarily age 20 years. Equations 1 and 2 vary according to the proportion of women starting OC use before age 16–19 years:

| | |
|---|---|
| $0.31\,OR^* + 0.69\,OR = 1.0$ (1) | 0.31: proportion starting < 20 years in cohorts 1945–50 |
| $0.72\,OR^* + 0.28\,OR = 2.0$ (2) | 0.72: proportion starting < 20 years in cohorts 1951–67 |
| $0.18\,OR^* + 0.82\,OR = 1.0$ (1′) | 0.18: proportion starting < 19 years in cohorts 1945–50 |
| $0.61\,OR^* + 0.39\,OR = 2.0$ (2′) | 0.61: proportion starting < 19 years in cohorts 1951–67 |
| $0.07\,OR^* + 0.93\,OR = 1.0$ (1″) | 0.07: proportion starting < 18 years in cohorts 1945–50 |
| $0.41\,OR^* + 0.59\,OR = 2.0$ (2″) | 0.41: proportion starting < 18 years in cohorts 1951–67 |
| $0.02\,OR^* + 0.98\,OR = 1.0$ (1‴) | 0.02: proportion starting < 17 years in cohorts 1945–50 |
| $0.22\,OR^* + 0.78\,OR = 2.0$ (2‴) | 0.22: proportion starting < 17 years in cohorts 1951–67 |
| $0.00\,OR^* + 1.00\,OR = 1.0$ (1⁗) | 0.00: proportion starting < 16 years in cohorts 1945–50 |
| $0.06\,OR^* + 0.94\,OR = 2.0$ (2⁗) | 0.06: proportion starting < 16 years in cohorts 1951–67 |

Equations 1 and 2 result in OR = 0.24 and OR* = 2.68, with a ratio of 11.2
Equations 1′ and 2′ result in OR = 0.58 and OR* = 2.90, with a ratio of 5.0.
Equations 1″ and 2″ result in OR = 0.79 and OR* = 3.73, with a ratio of 4.7.
Equations 1‴ and 2‴ result in OR = 0.90 and OR* = 5.90, with a ratio of 6.5.
Equations 1⁗ and 2⁗ result in OR = 1.0 and OR* = 17.7, with a ratio of 17.7.

© Cancer Research Campaign 1997

All these estimates are necessarily unprecise, being based on small numbers. They could be used in connection with speculations regarding the critical age at first use, arguing that a very high ratio between OR* and OR is unlikely, thus making age 18 years an appealing choice.

Finally, we do not completely agree with Dr Tómasson when he claims that the two Icelandic studies and the results of the Collaborative Group on Hormonal Factors in Breast Cancer (1996) all show that use of OC has very little impact on the risk for breast cancer. We feel that there is still a question mark concerning the effects of OC use at young age. Our study, as well as the one by the Collaborative Group give rise to some concern about this matter. The study of the Collaborative Group also found an increased risk in young users and, to quote the paper, 'The available data for use beginning before age 20 indicate that there is no substantial increase of breast cancer risk in this subgroup more than 5 years after cessation of use, but virtually all the existing information relates to women younger than 45. In the next decade women who began use as teenagers will reach their late 40s and early 50s, when breast cancer is more common. When the new data on the long-term effects of early use become available it will be necessary to re-examine the worldwide evidence'.

*L Tryggvadóttir, H Tulinius and GB Gudmundsdóttir*

*Epidemiological Unit, Icelandic Cancer Registry,*

*Icelandic Cancer Society, PO Box 5420, IS-125 Reykjavik, Iceland*

**REFERENCES**

Ambrosone CB, Freudenheim JL, Graham S, Marshall JR, Vena JE, Brasure JR, Michalek AM, Laughin R, Nemoto T, Gillenwater KA, Harrington AM and Shields PG (1996) Cigarette smoking, *N*-acetyltransferase 2 genetic polymorphisms, and breast cancer risk. *JAMA* **276**: 1494–1501

Collaborative Group on Hormonal Factors in Breast Cancer (1996) Breast cancer and hormonal contraceptives: collaborative reanalysis of individual data on 53,297 women with breast cancer and 100,239 women without breast cancer from 54 epidemiological studies. *Lancet* **347**: 1713–1727

Tómasson H and Tómasson K (1996) Oral contraceptives and risk of breast cancer. A historical prospective case–control study. *Acta Obstet Gynecol Scand* **75**: 157–161

Tryggvadóttir L, Tulinius H and Gudmundsdóttir GB (1997) Oral contraceptive use at a young age and the risk of breast cancer. An Icelandic, population-based cohort study of the effect of birth year. *Br J Cancer* **75**: 139–143